# Antiproliferative effect of somatostatin analogs in advanced gastro-entero-pancreatic neuroendocrine tumors: a systematic review and meta-analysis

**DOI:** 10.18632/oncotarget.16686

**Published:** 2017-03-29

**Authors:** Elettra Merola, Francesco Panzuto, Gianfranco Delle Fave

**Affiliations:** ^1^ Department of Digestive and Liver Diseases, Sapienza University, Sant'Andrea Hospital, Rome, Italy

**Keywords:** gastro-entero-pancreatic neuroendocrine tumors, somatostatin analogs, antiproliferative effect, meta-analysis

## Abstract

A meta-analysis has systematically investigated the antineoplastic efficacy and safety of somatostatin analogs (SSAs) in advanced gastro-entero-pancreatic neuroendocrine tumors (GEP-NETs). Randomized controlled trials (RCTs) reporting the hazard ratio (HR) for disease progression (DP) were evaluated. Response rate and risk ratio (RR) for adverse events were also analyzed. A total of 289 patients (143 receiving SSAs *vs*. 146 placebo) were evaluated from two RCTs. A significant benefit from SSAs in terms of disease control was observed (HR 0.41, 95% CI: 0.29 to 0.58, *P* < 0.01; *I*20%), response rate being 58.0% *vs*. 32.2%, respectively. The occurrence of adverse events significantly differed from the placebo arm only in terms of biliary stones (RR 3.79, 95% CI: 1.28 to 11.17, *P* = 0.02; *I*20%). In conclusion, SSAs showed an antiproliferative effect in advanced GEP-NETs, with a good safety profile.

## INTRODUCTION

Gastro-Entero-Pancreatic Neuroendocrine Tumors (GEP-NETs) have an incidence of approximately 3 to 5 per 100,000 person/year and a prevalence of 35 per 100,000 [1–2]. When locally advanced or metastatic (70% at diagnosis), 5-year overall survival (OS) accounts for 35% to 55%, and mortality rate is 2-fold higher than lower stages [3–7]. In these cases, surgery with radical intent is not feasible, and medical treatments should be chosen according to tumor features [8–9].

Ninety percent of GEP-NETs express somatostatin receptors (SSTRs) on cell surface, and may benefit from somatostatin-based treatments: “cold” somatostatin analogs (SSAs) or peptide receptor radionuclide therapy (PRRT) [10–13]. SSAs (i.e., octreotide, lanreotide, pasireotide) were initially adopted to inhibit the release of neuropeptides or amines responsible for clinical syndromes. Their effect on tumor proliferation was subsequently suggested by uncontrolled studies, extending their use to non-functioning cases [10, 14–17]. They represent a frequently adopted first-line option in advanced GEP-NETs, with good toxicity profile. Two double-blind randomized controlled trials (RCTs) enrolled unresectable GEP-NETs to investigate the antineoplastic role of octreotide and lanreotide respectively [18–21]. However, there are no quantitative data syntheses of their efficacy in terms of disease control (also considering the recently published long-term follow-up data), nor of their safety profile. In fact, previous systematic reviews (SRs) were published before the CLARINET trial and its long-term follow-up data [19, 21], and thus results need to be updated. In addition, these reviews did not perform any meta-analyses, as they also included case series, case control studies or retrospective series [9, 22–26].

The aim of this paper is to measure the antiproliferative effect and safety of SSAs compared to placebo, in patients with advanced GEP-NETs, through an updated systematic review and a meta-analysis of available RCTs.

## RESULTS

### Search results

**Figure 1:**
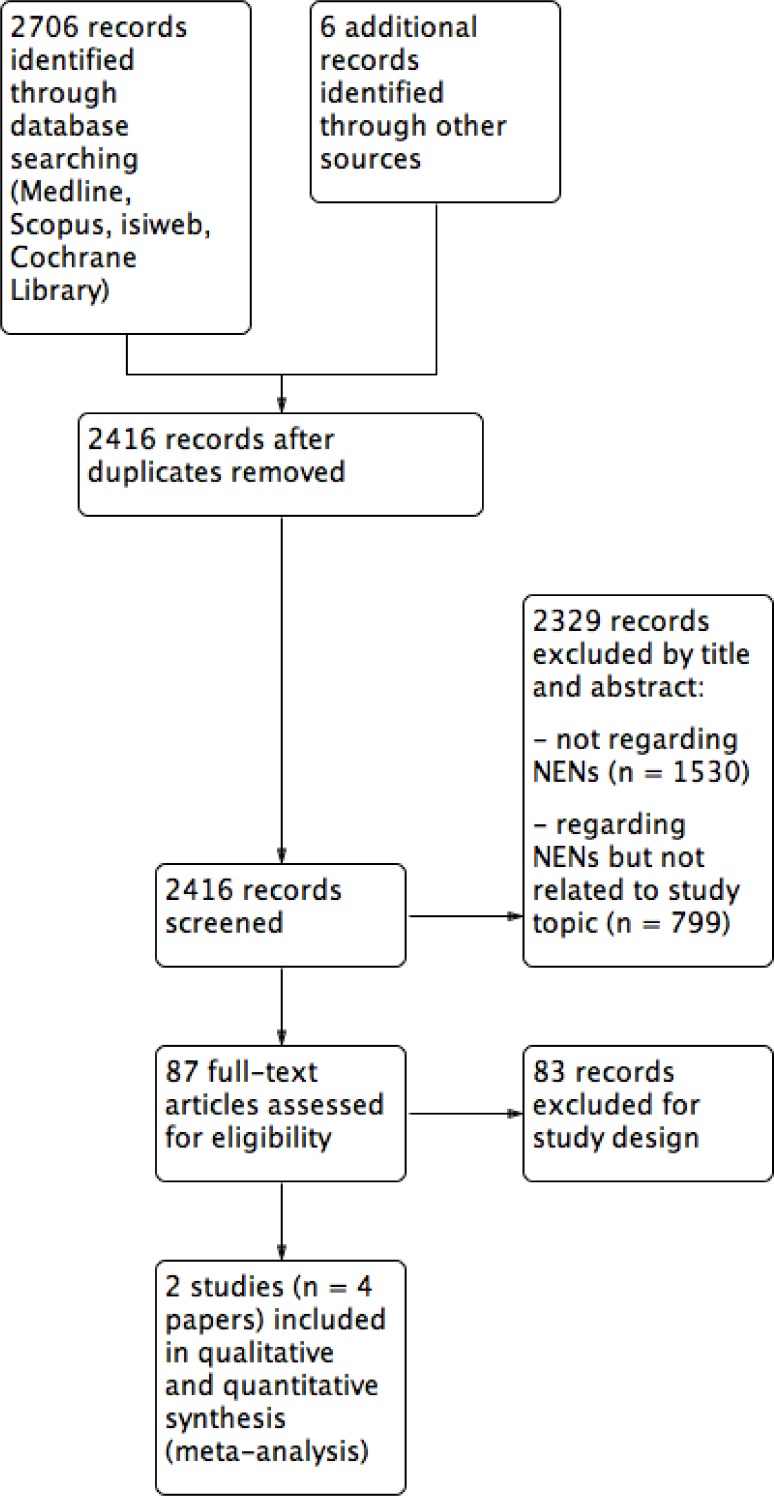
Study selection sequence to perform the meta-analysis

Database searches yielded the following 2706 references: 247 from Medline, 2293 from Scopus, 34 from Isi web of science, and 132 from the Cochrane Library (Figure 1). Six additional references were retrieved from grey literature. Exclusion of duplicates left 2416 references, and 2329 of these did not satisfy inclusion criteria. Full texts were examined in 87 publications concerning the use of SSAs in NETs, and 83 were excluded due to the study design. Thus, only two RCTs (4 papers) were considered for qualitative and quantitative analyses [18–21], with a total overall population of 289 cases (143 in the SSA arm and 146 in the placebo arm). A direct comparison of the two study methods is reported in Table 1, while patients’ features are summarized in Supplementary Table 1.

**Table 1:** Patients and methods: comparison between the two studies included in the meta-analysis

Features	PROMID [18]	CLARINET [19]
Tumor primary site	Midgut (some unknown believed to be midgut; not specified if sporadic)	Gatroenteropancreatic and unknown (all sporadic)
Stage	Unresectable metastatic or locally advanced	Unresectable metastatic or locally advanced, or refusing surgery
Histopathology	Well-differentiated; mostly ki67 ≤ 2%	Well- or moderately-differentiated; ki67 < 10%
Disease status	Not stated in the methodology	Stable (96%) and progressive disease (4%)
Clinical syndrome included	Carcinoid	Zollinger-Ellison (well-controlled)
Performance status	Karnofsky scale ≥ 60%	WHO scale ≤ 2
Somatostatin receptors expression	Not specified	Octreoscan® positivity (grade 2-4 Krenning Scale)
Excluding comorbidities	Other cancers	Genetic syndromes (i.e. MEN), other cancers (unless disease-free for > 5 years)
Previous treatments	Naïve patients (only SSAs for ≤ 4 weeks allowed)	Mostly (84%) naïve patients (only SSAs for ≤ 2 weeks allowed, > 6 months previously)
Study drug	Octreotide LAR 30 intramuscular every 28 days	Lanreotide 120 mg deep subcutaneous injection every 28 days
Centers included	18 academic German centers	14 countries (Europe, USA, India)
Primary outcome	TTP	PFS
Progression evaluation	WHO criteria	RECIST criteria (version 1.0)
Study duration	Until disease progression	96 weeks (24 drug administrations)
Liver burden categories	0% / ≤ 10% / 10%-25% / 25%-50% / > 50%	≤ 25% / > 25%
Toxicity evaluation	WHO criteria or National Cancer Institute Common Toxicity Criteria (version 2.0)	“Medical Dictionary for Regulatory Activities”, (version 16.0)
Study update	Long-term follow-up of both placebo and SSA arm (OS analysis)	Single-arm, non randomized, multicenter study: cross-over for placebo arm, continuation of lanreotide for SSA arm stable at 96 weeks (PFS and safety analysis), OS missing

Abbreviations: WHO: World Health Organization; MEN: Multiendocrine Neoplasia; SSAs: Somatostatin Analogs; LAR: long-acting release; TTP: Time to Progression; PFS: Progression-free Survival; RECIST: Response Evaluation Criteria in Solid Tumors; OS: Overall Survival;

### Risk of bias

The evaluation of the risk for bias for both studies is reported in Supplementary Figure 1.

With regard to the PROMID study [18], sequence generation and blinding were adequate, while allocation concealment was unclear. In fact, after centralized randomization, the different study centers were informed of patient's assignment to the treatment or placebo groups. Then, a solution of octreotide or sodium chloride was administered by members of the hospital staff not involved in the trial, but the method for concealment was not specified.

Conversely, the CLARINET study [19] showed a low risk of all evaluated biases.

### Disease progression (DP)

**Figure 2:**
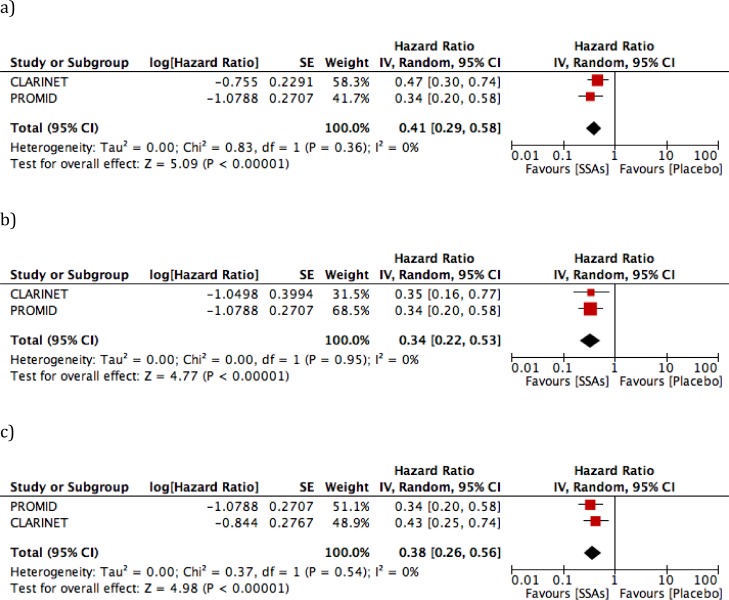
Forest plots for progression-free survival (PFS) **a**. overall population; **b**. midgut tumors; **c**. G1 tumors.

Considering the overall population, 158/289 (54.7%) DPs were observed (Table 2). Comparison of treatment *vs*. placebo arms showed rates of 58/143 (40.5%) and 100/146 (68.5%), respectively. The benefit from SSAs use was confirmed by forest plot (HR 0.41, 95% CI: 0.29 to 0.58, *P* < 0.01; Figure 2a), and the study results showed no statistically significant heterogeneity (*I*2 = 0%, *P* = 0.36).

**Table 2:** Antiproliferative effect: comparison between the two studies included in the meta-analysis

	**PROMID** [18]		**CLARINET** [19]	
**Overall population**	**SSA (*n* = 42)**	**Placebo (*n* = 43)**	**SSA (*n* = 101)**	**Placebo (*n* = 103)**
Disease control rate * CR, *n* (%) PR, *n* (%) SD, *n* (%)	0 (0) 1 (2.4) 15 (35.7)	0 (0) 1 (2.3) 2 (4.7)	0 (0) 2 (1.9) [36] 65 (64.3)	0 (0) 0 (0) 44 (42.7)
DP, n (%)	26 (61.9)	40 (93.0)	32 (31.7)	60 (58.2)
Primary outcome TTP, [months; median (95% CI)] PFS, [months; median (95% CI)]	14.3 (11.0 to 28.8) -	6.0 (3.7 to 9.4) -	- n.r.	- 18 (12.1 to 24.0)
**Midgut primary site**	**SSA (*n* = 42)**	**Placebo (*n* = 43)**	**SSA (*n* = 33)**	**Placebo (*n* = 40)**
DP, *n* (%)	26 (61.9)	40 (93.0)	8 (24.2)	21 (52.5)
Primary outcome TTP, [months; median (95% CI)] PFS, [months; median (95% CI)]	14.3 (11.0 to 28.8) -	6.0 (3.7 to 9.4) -	- n.r.	- 21.1 (17 to NC)
**Pancreatic primary site**	**SSA (*n* = 0)**	**Placebo (*n* = 0)**	**SSA (*n* = 42)**	**Placebo (*n* = 49)**
DP, *n* (%)	0 (0)	0 (0)	18 (42.8)	31 (63.3)
Primary outcome TTP, [months; median (95% CI)] PFS, [months; median (95% CI)]	- -	- -	- n.r.	- 12.1 (9.4 to 18.3)
**G1 cases**	**SSA (*n* = 42)**	**Placebo (*n* = 43)**	**SSA (*n* = 69)**	**Placebo (*n* = 72)**
DP, *n* (%)	26 (61.9)	40 (93.0)	19 (27.5)	40 (55.5)
Primary outcome TTP, [months; median (95% CI)] PFS, [months; median (95% CI)]	14.3 (11.0 to 28.8) -	6.0 (3.7 to 9.4) -	- n.r.	- 18.3 (12.7 to 24.0)
**G2 cases**	**SSA (*n* = 0)**	**Placebo (*n* = 0)**	**SSA (*n* = 32)**	**Placebo (*n* = 29)**
DP, *n* (%)	-	-	13 (40.6)	19 (65.5)
Primary outcome TTP, [months; median (95% CI)] PFS, [months; median (95% CI)]	- -	- -	- n.r.	- 12.1 (9.0 to 18.0)

* Best response assessed during the study

Abbreviations: SSA: Somatostatin Analog; CR: Complete Response; PR: Partial Response; SD: Stable Disease; DP: Disease Progression; TTP: Time to Progression; PFS: Progression-free Survival; CI: Confidence Interval; n.r.: not reached; NC: not calculable

Focusing on midgut tumors, cases enrolled in the PROMID study [18] as “unknown primary site” were also included in the quantitative analysis, when suspected to be of small bowel origin in the trial. DP was observed in 34/75 (45.3%) midgut NETs treated with SSAs *vs*. 61/83 (73.5%) in the untreated arm. The meta-analysis excluded a significant heterogeneity between the RCTs (*I*2 = 0%, *P* = 0.95) and confirmed the efficacy of SSAs in disease control, with HR 0.34 (95% CI: 0.22 to 0.53, *P* < 0.01; Figure 2b).

With regard to G1 cases, DP was observed in 45/111 (40.5%) in the SSA treated arm *vs*. 80/115 (69.6%) in the placebo arm. No significant heterogeneity was observed between the 2 studies (*I*2 = 0%, *P* = 0.54), and benefit from SSA treatment was confirmed by the meta-analysis (HR 0.38, 95% CI: 0.26 to 0.56, *P* < 0.01; Figure 2c).

Only the CLARINET study [19] evaluated PFS in pancreatic and G2 NETs (with ki67 < 10%), showing a trend favorable to lanreotide in these subgroups but without statistical significance for pancreatic primary site. Two cases from the PROMID trial [18] were identified, after enrollment, as histologically ki67 > 2%, but it was not possible to exclude their outcome from the overall population.

A sub-analysis by liver tumor load was not feasible, since data presented in the two RCTs referred to different cut-off values (Table 1).

The trials have been recently updated in two different papers [20–21], reporting a long-term analysis of PFS and safety. The PROMID study [18] was terminated early as soon as octreotide efficacy *vs*. placebo was proved; however, long-term follow-up (median 96 months) data was collected. Post-study treatment was chosen by local investigators at each single center [20]. Median time of exposure to octreotide long-acting release (LAR) was 70.5 months (1.2 to 140.2) for cases randomly assigned to SSA arm, and 53.1 (0.1 to 127) for untreated patients who then started octreotide LAR. Four out of 42 patients in the SSA arm still had SD, while all placebo cases reported DP or death.

With regard to the CLARINET trial [19], it continued as a single-arm, non-randomized, multicenter study [21]. Eighty-eight patients with SD at the end of the primary study or reporting DP in the placebo arm continued or switched to lanreotide: 41 from the SSA arm (LAN-LAN), and 47 previously untreated (PBO-LAN). The total time of lanreotide exposure was 40 months (range: 26 to 74.3) for the first group and 18.1 (range: 1 to 49.9) when started at SD, 13 (2 to 52) if initially progressive, for the second one. Considering the overall LAN-LAN population, PD rate was 45/101 (44.5%), with 23 cases still continuing the study, and a median PFS of 32.8 months (95% CI: 30.9 to 68.0). The PBO-LAN group showed a PD rate of 20/47 (42.5%), with 18 patients continuing the open-label study. When patients starting lanreotide at DP were considered, new median PFS was 14.0 months in 17/32 (53.1%) cases.

### Disease control rate

Disease control was observed in 83/143 (58.0%) cases with SSAs and 47/146 (32.2%) with placebo (Table 2). No CRs were observed.

### Overall survival

When the original trials [18–19] were considered, 7/143 (4.9%) tumor-related deaths were reported in the treatment arm *vs*. 9/146 (6.2%) in the placebo arm. A meta-analysis according to the “generic inverse variance method” could not be performed, as the CLARINET trial [19] did not report the HR value for OS. With regard to long-term follow-up data [21], HR was still missing, and the cross-over design would be a further limit to a quantitative analysis.

Instead, the long-term follow-up from PROMID study [20] showed 38/85 (44.7%) NET-related deaths: 17 (40.5%) in the treatment arm and 21 (48.8%) in the control arm. Median OS was 84.7 and 83.7 months, respectively (*P* = 0.51). There was a statistically significant difference in terms of OS when the two arms were compared after stratification by liver tumor load (cut-off: 10%) and primary tumor resection.

### Safety

Table 3 summarizes safety data, and reports the adverse events recorded in both RCTs [18–21]. It is impossible to calculate their total amount, as they were not reported clearly neither in the original PROMID study [18] nor in its long-term analysis [20].

**Table 3:** Toxicity: comparison between the two studies included in the meta-analysis

	PROMID [18]		CLARINET [19]	
Drug-related adverse events	SSA (*n* = 42)	Placebo (*n* = 43)	SSA (*n* = 101)	Placebo (*n* = 103)
Death, *n* (%)	0 (0)	0 (0)	0 (0)	0 (0)
Total number, *n* (%)	-	-	50 (49.5)	29 (28.1)
SAE, *n* (%)	11 (26.2)	10 (23.2)	3 (2.9)	1 (0.9)
Severe, *n* (%)	19 (45.2)	11 (25.6)	26 (25.7)	32 (31.1)
Treatments discontinuation, *n* (%)	5 (11.9)	0 (0)	1 (0.9)	0 (0)
Biliary stones, *n* (%)	5 (11.9)	1 (2.3)	10 (9.9)	3 (2.9)

Abbreviations: SSA: Somatostatin Analog; SAE: Serious Adverse Events

**Figure 3:**
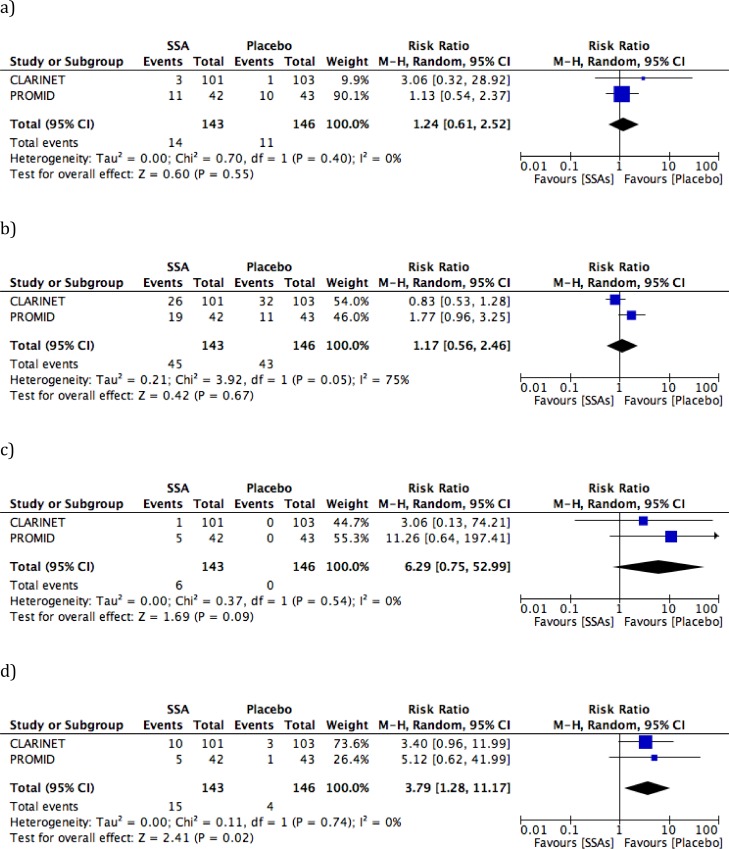
Forest plots for toxicity **a**. serious adverse events (SAE); **b**. adverse events of severe grade; **c**. treatment discontinuation; **d**. biliary stones.

The serious adverse events (SAE) meta-analysis showed no statistical significance neither in terms of heterogeneity (*I*2 = 0%, *P* = 0.40; Figure 3a) nor in terms of difference between the two arms (*P* = 0.55). The events were more frequent with octreotide than lanreotide (26.2% *vs*. 2.9%, respectively), but in the PROMID trial [18] they were not specified as being “drug-related”. As far as “severe events” are concerned (Figure 3b), a correct quantitative analysis was not feasible, due to significant heterogeneity (*I*2 = 75%, *P* = 0.05). In the CLARINET study [19] adverse events were more frequent in the placebo arm than in the lanreotide arm (31.1% *vs*. 25.7%, respectively). In general, the incidence of adverse events was higher than that reported in the long-term analysis [21], except for diarrhea in the PBO-LAN group.

The long-term results of the PROMID [20] study did not include safety data, as safety was not considered as an endpoint of the updated protocol.

A trend towards significance was observed in the meta-analysis in terms of treatment discontinuation (*P* = 0.09; Figure 3c), while a statistically significant difference (RR 3.79, 95% CI: 1.28 to 11.17, *P* = 0.02; Figure 3d) was observed for the occurrence of biliary stones: 10.5% in the SSA *vs*. 2.7% in the placebo arm, respectively, with no heterogeneity recorded (*I*2 = 0, *P* = 0.74).

## DISCUSSION

The present study confirms the antiproliferative effect of SSAs in advanced GEP-NETs, by showing a statistically significant difference in PFS in comparison to placebo. In detail, the risk of DP using SSAs decreases by 41% (HR), independently from tumor primary site and G grading.

Previous SRs had reported that SSAs were effective in controlling GEP-NETs proliferation [22–26], but they did not perform any meta-analyses and did not include the data on long-term follow-up of the PROMID and the CLARINET trials [18–21].

Although in a smaller population than in the CLARINET study [19], the PROMID trial [18] showed a clear benefit from octreotide LAR use, reaching the study goal before the expected time and thus being early terminated. However, DP rate in the treatment arm was 2-fold higher (61.9%) than in the CLARINET trial [19] (31.7%), although the latter also included pancreatic and hindgut primary sites, a proportion of G2 cases and a higher liver tumor burden. This gap was also observed between the placebo arms, with rates of 93.0% and 58.2%, respectively. This difference in the number of events is certainly related to the different disease status at study entry (defined as SD only in the CLARINET [19, 21] trial), and probably also due to the different criteria used to assess PD in the studies (“World Health Organization” and “Response Evaluation Criteria in Solid Tumors”) [27–28]. Furthermore, the PROMID trial [18] included 4 cases with Ki67 > 2%, and 10 with negative uptake at Octreoscan®. Conversely, the CLARINET trial [19] had a more accurate method, requiring a biopsy within 6 months before enrollment in case of progression, suspicion or neoplastic comorbidity.

In both the PROMID and the CLARINET studies, Kaplan-Meyer curves showed a PFS difference after about 3 months of overlapping. Their results showed a median time to progression (TTP) of 14.3 months with octreotide *vs*. a “not reached” median PFS with lanreotide, respectively. These results could be compared to the “post-hoc” analysis of the RADIANT-2 study [29], which showed the long-term analysis of the control arm (placebo + LAR 30) in patients with advanced progressive GEP-NETs with carcinoid syndrome. Focusing on the 41 SSA-naïve cases, median PFS was 13.6 months, similar to the PBO-LAN group of the CLARINET extension study [21] (14.0 months) and to the PROMID study [18] (14.3 months). Thus, three different trials are consistent with a median PFS of about 14 months for first-line SSAs used in progressive, advanced GEP-NETs. A recent study has reported a longer median TTP (37 months) when adopting octreotide at first line in advanced NETs [30]. On the contrary, the NETTER-1 trial [13] investigating the efficacy of (^177^Lu)-DOTATATE in advanced, progressive midgut NETS after failure of standard dose octreotide LAR, showed that “high dose octreotide” (60 mg every 4 weeks) adopted in the control arm was less effective than LAR 30 mg every 4 weeks used in the PROMID trial [18] (8.4 *vs*. 14.3 months, respectively). However the comparison among these studies is not feasible, due to several differences in terms of study design and enrolled populations.

As far as mortality rates between the PROMID [18] and the CLARINET study [19] are concerned, they were higher in the former study (14.1 % *vs*. 1.9%, respectively), suggesting a different tumor behavior in the two populations. In the CLARINET study, OS was lower for pancreatic and G2 cases than for midgut and G1, as expected from literature. However, a meta-analysis could not be performed as HR for OS was not reported in the CLARINET trial [19, 21], which had also a cross-over design that might influence the quantitative analysis. Thus, conclusions about the effects of SSAs on OS cannot be drawn on the basis of these two RCTs. However, an observational study presented at the ASCO Annual Conference 2015 [31] suggested PFS to be associated with OS. In detail, in 140 metastatic NETs treated with SSAs and followed-up for a median time of 7.6 years, OS was shorter for progressive neoplasms than for cases with SD, supporting the relevance of PFS as an endpoint in NETs clinical trials.

With regard to safety, SSAs showed a good profile, with no drug-related deaths and most side effects being of low to moderate grade. Forest plot for “severe events” showed a significant heterogeneity, probably due to a different classification of toxicity used in the included RCTs (Figure 3). The meta-analyses for SAEs and discontinuation due to toxicity showed no statistically significant difference between treatment and placebo arms, while the occurrence of biliary stones was considerably higher in the SSA arm (10.5%) than in untreated patients (2.7%). This result might support the role of prophylactic cholecystectomy in metastatic patients facing resection of primary tumors.

Octreotide seemed to be responsible for severe toxicity more frequently than lanreotide (45.2% *vs*. 25.7%, respectively). However, in the PROMID trial [18, 20], these events were not clearly specified as being “drug-related”, with a subsequent possible overestimation. On the other side, the CLARINET study, although offering a more detailed and updated description of adverse events, showed a higher occurrence in the placebo than in the treatment arm (31.1% *vs*. 25.7%, respectively) [19, 21]. This difference might be related to both the good safety profile of SSAs and a possible manifestation of cancer-related symptoms.

A clear limitation of the present study is the inclusion of only two RCTs in the meta-analysis, meeting just the minimal number of studies required for this methodology. This issue can not currently be solved as no other RCTs focusing on the efficacy of SSAs *vs*. placebo in tumor control are expected. Thus, although several SRs with the same aim have been previously published [22–26], the present study strengthens their results through a meta-analysis. This evidence-based method can be hardly applied to the other published trials for NETs, as they compare different treatment arms and analyze heterogeneous populations.

Another possible bias of this study is the comparison of two GEP-NETs populations with some inconsistencies in the inclusion criteria (Table 1). In fact, although the forest plots for the primary outcome excluded a statistical heterogeneity (*I*2 = 0%), there is a difference in the tumor behavior related to the disease status at study entry (not stated in the PROMID trial but probably including PD *vs*. mainly SD in the CLARINET trial). The primary outcome also was differently expressed in these RCTs (TTP *vs*. PFS rates, respectively), and this might represent a further limit for data interpretation.

In conclusion, the present meta-analysis confirms that SSAs have an antiproliferative effect in advanced GEP-NETs, reducing DP risk by 41%, with a good safety profile. New trials evaluating SSAs efficacy in pancreatic and G2 NETs are needed to validate results also in these categories of patients.

## MATERIALS AND METHODS

### Inclusion criteria and outcomes

Only RCTs comparing SSAs to placebo in terms of antiproliferative effect were considered in this review. Patients were included if adults (age ≥ 18 years) and affected by advanced (locally inoperable or metastatic), sporadic GEP-NETs. Disease had to be histologically proven and measurable through computed tomography or magnetic resonance imaging. Exclusion criteria included concomitant antineoplastic treatments.

All available SSAs (i.e., octreotide, lanreotide, pasireotide) at all dosages were taken into consideration.

The primary outcomes of this review were: 1) hazard ratio (HR) for progression-free survival (PFS); 2) disease control rates (according to criteria adopted in each study): partial response (PR), complete response (CR) or stable disease (SD).

The secondary outcomes were: 1) HR for OS; 2) safety in terms of risk ratio (RR) for adverse events.

Subanalyses according to primary sites, G grading [32–33] and hepatic tumor burden were planned at protocol stage.

### Search method

Computerized searches were performed to identify all published and unpublished RCTs satisfying inclusion criteria, without applying any filters in terms of language.

The databases searched were Medline, Scopus, ISI web of knowledge and Cochrane Database of Systematic Reviews, with search strategy last updated on 27^th^ March 2016. For “disease condition”, the following terms were used: (((neuroendocrine OR endocrine) AND (tumor OR tumour OR cancer OR carcinoma OR neoplasm*)) OR carcinoid OR “islet cell carcinoma”) AND (digestive OR gastrointestinal OR gastroenteropancreatic OR pancreatic OR intestinal OR midgut OR bowel). The search also included the following terms for “therapy”: lanreotide OR octreotide OR somatostatin OR pasireotide. “Publication type” was selected by adding: randomized controlled trial, controlled clinical trial, randomized, placebo, randomly, trial, or groups.

The Endnote program (Endonte X4, Bld 6695) was used for study selection and reference management. Grey literature was also considered, including hand-search on conference abstract books from the following journals: the American Gastroenterological Association (AGA), the European Society for Medical Oncology (ESMO), the American Society of Clinical Oncology (ASCO), the European Neuroendocrine Tumors Society (ENETS), and the North American Neuroendocrine Tumor Society (NANETS). The clinicaltrials.gov website was also searched, as well as the reference lists of all the selected papers.

Study selection was in agreement with PRISMA Guidelines [34]. In the first instance, titles and abstracts were screened to evaluate whether the publications responded to the inclusion criteria. Then, complete full texts were reviewed, and papers judged useful for qualitative and quantitative analyses were included.

### Data extraction

Two independent reviewers (E.M., F.P.) carried out the search, study selection and data extraction. In case of disagreement, the opinion of a third reviewer (G.D.F.) was requested. Excluded studies and the reasons for exclusion were recorded. In case of duplicate publications, the most updated version was considered. The following features were recorded for each trial:

· first author and year of publication

· number of patients included in both arms

· time from diagnosis to enrollment

· reported reason for withdrawal

· tumor primary sites

· ki67/ G grading [32–33]

· tumor liver burden

· treatment (drug name and dosage)

· HR and RR values

· CR, PR, SD and DP rates

· tumor-related death cases

· adverse events.

We used published data without contacting study authors.

### Risk of bias

Study quality was evaluated by two authors (E.M. and F.P.) independently, following the instructions reported on the Cochrane Handbook for Systematic Reviews of Intervention [35]. Disagreements were resolved by a third author (G.D.F.).

RCTs classified as adequate in sequence generation, allocation concealment, blinding, incomplete data outcomes and selective reporting were defined as being at “low risk of bias”.

### Statistical method

Meta-analyses were performed according to the Cochrane Collaboration methodology [35] using the software package RevMan 5.2.5 (RevMan 2012). For outcomes such as HR, the “generic inverse variance method” was used, while for outcomes such as RR, Mantel-Haenszel method was used, with 95% confidence interval (95% CI). Both random-effects and a fixed-effect model were used, and they were both reported in case of significant discrepancy; otherwise, the fixed-effect model was used. Heterogeneity was assessed by Chi-squared test with significance set at *P* value < 0.10, and the quantity of heterogeneity measured by *I*2. We considered an *I*2 of ≥ 30% as representative of heterogeneity. The analysis was planned to be based on intention-to-treat (ITT) population.

## SUPPLEMENTARY MATERIALS FIGURES AND TABLES





## Abbreviations

GEP-NETs: gastro-entero-pancreatic neuroendocrine tumors
OS: overall survival
SSTRs: somatostatin receptors
SSAs: somatostatin analogs (SSAs)
PRRT: peptide receptor radionuclide therapy
RCTs: randomized controlled trials
SRs: systematic reviews
HR: hazard ratio
PFS: progression-free survival
PR: partial response
CR: complete response
SD: stable disease
RR: risk ratio
CI: confidence interval
ITT: intention-to-treat.
