# Supplementary material for: Antiproliferative effect of somatostatin analogs in advanced gastro-entero-pancreatic neuroendocrine tumors: a systematic review and meta-analysis
Source: Oncotarget. 2017 Mar 29;8(28):46624–34. doi: 10.18632/oncotarget.16686 (PMC5542298; doi:10.18632/oncotarget.16686)
Supplement: Supplementary file 1 [file oncotarget-08-46624-s001.pdf]

Antiproliferative effect of somatostatin analogs in advanced gastro-entero-pancreatic neuroendocrine tumors: a systematic review and meta-analysis

Supplementary Material

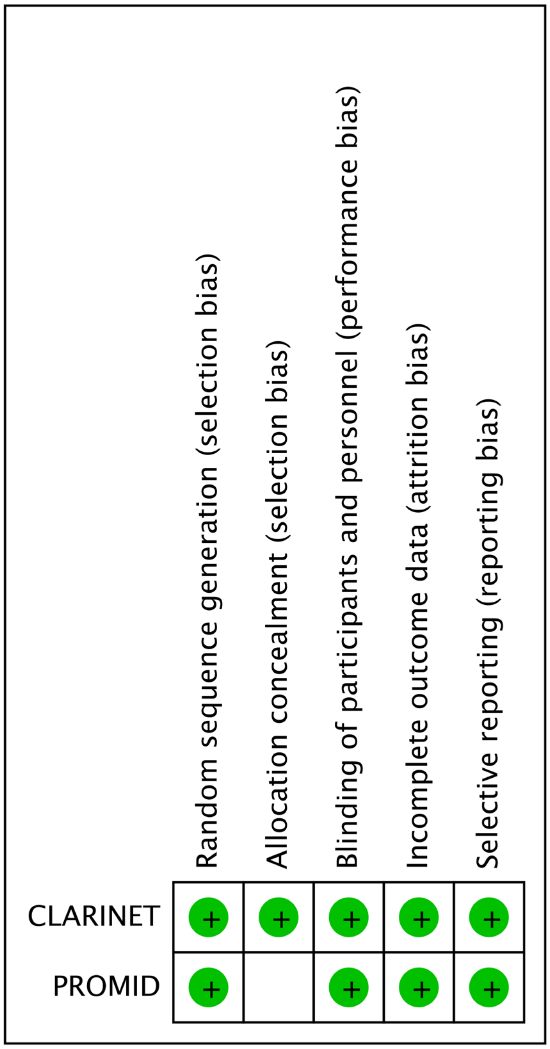

Supplementary Figure 1: Risk of bias for the included studies. Low risk is indicated by the "green light", while the "red light" corresponds to high risk and none to unclear risk.

For Supplementary Table 1 see in Supplementary Files
