# Supplementary material for: Antiproliferative effect of somatostatin analogs in advanced gastro-entero-pancreatic neuroendocrine tumors: a systematic review and meta-analysis
Source: Oncotarget. 2017 Mar 29;8(28):46624–34. doi: 10.18632/oncotarget.16686 (PMC5542298; doi:10.18632/oncotarget.16686)
Supplement: Supplementary file 2 [file oncotarget-08-46624-s002.pdf]

Antiproliferative effect of somatostatin analogs in advanced gastro-entero-pancreatic neuroendocrine tumors: a systematic review and meta-analysis

Supplementary Table 1. Patients' presentation: comparisons between the two studies included in the meta-analysis.

	**PROMID [18]**		**CLARINET [19]**	
**Features**	**SSA (*n* = 42)**	**Placebo (*n* = 43)**	**SSA (*n* = 101)**	**Placebo (*n* = 103)**
Tumor primary site				
Midgut, *n* (%)	42 (100)	43 (100)	33 (32.7)	40 (38.8)
Pancreas, *n* (%)	0 (0)	0 (0)	42 (41.6)	49 (47.6)
Others or unknown, *n* (%)	not reported	not reported	26 (25.7)	14 (13.6)
Withdrawn, *n* (%)	16 (38.1)	4 (9.3)	18 (17.8)	21 (20.4)
Age, median (range)	63.5 (38-79)	61 (39-82)	63.3 (53.5-73.1)	62.2 (51.1-73.3)
Gender (male), *n* (%)	20 (47.6)	23 (53.5)	53 (52.5)	54 (52.4)
Time since diagnosis, median (range)	7.5 (0.8-271.7)	3.3 (0.8-109.4)	13.2 (range not reported)	16.5 (range not reported)
Functional syndrome				
Carcinoid, *n* (%)	17 (40.5)	16 (37.2)	0 (0)	0 (0)
Zollinger-Ellison, *n* (%)	0 (0)	0 (0)	2 (1.9)	2 (1.9)
Resection of primary tumor, *n* (%)	29 (69.1)	27 (62.8)	40 (39.6)	39 (37.8)

Prior medical treatments, *n* (%)	0 (0)	0 (0)	16 (15.8)	16 (15.5)
G grading *				
G1, n (%)	41 (97.6)	40 (93.0)	69 (68.3)	72 (71.3)
G2, n (%)	1 (2.4)	3 (7.0)	32 (31.7)	29 (28.7)
Somatostatin receptors expression				
Positive, *n* (%)	32 (76.2)	31 (72.1)	101 (100.0)	103 (100.0)
Negative, *n* (%)	4 (9.5)	6 (14.0)	0 (0)	0 (0)
Unknown, *n* (%)	6 (14.3)	6 (13.9)	0 (0)	0 (0)
Liver tumor load				
≤ 25%, *n* (%)	35 (83.3)	34 (79.1)	62 (61.4)	75 (72.8)
> 25%, *n* (%)	7 (16.7)	9 (20.9)	39 (38.6)	28 (27.2)

* Ki67 missing in 2 patients of the CLARINET study

SSAs: Somatostatin Analogs
